# Biogenic synthesis and antimicrobial properties of rare-earth element nanoparticles using *Moringa oleifera*

**DOI:** 10.1039/d5ra04558d

**Published:** 2025-10-01

**Authors:** Nader Anis, Amira A. Gamal, Nada Emam, Mostafa Gomaa Fadl, A. I. L. Abd El Fatah

**Affiliations:** a Reactors Materials Department, Nuclear Materials Authority Cairo Egypt Naderanis@science.zu.edu.eg; b Chemistry of Natural and Microbial Products Department, Pharmaceutical and Drug Industries Research Institute, National Research Centre Giza Egypt

## Abstract

This research demonstrates a green biological method for producing mixed rare-earth oxide nanoparticles (multi-REO NPs) through *Moringa oleifera* leaf extract in water. A light rare-earth oxide (LREO) cake concentrate, containing mainly the oxides of La, Ce, Pr, and Nd, served as the precursor for the metal ions. The aqueous extract from *M. oleifera* leaves due to its rich phytochemical contents served dual functions as a bioreducing and capping agent. The formation of nanoparticles was verified through UV-vis spectroscopy, which displayed a characteristic absorption peak at 311 nm corresponding to CeO_2_. Fourier transform infrared (FTIR) spectroscopy confirmed the formation of the metal oxide core, with characteristic metal–oxygen (M–O) vibrational bands observed below 620 cm^−1^. XRD analysis revealed that the synthesized multi-REO NPs were predominantly nanocrystalline. Transmission electron microscopy (TEM) revealed nanoparticles with varied morphologies and an average diameter of 29.36 nm (ranging from 16 to 50 nm), further confirmed by dynamic light scattering (DLS), which showed a hydrodynamic diameter of 29.3 nm. Coupled scanning electron microscopy (SEM) and energy-dispersive X-ray spectroscopy (EDS) confirmed the presence of La, Ce, and Nd. The synthesized multi-REO NPs were nanocrystalline, exhibiting broad diffraction peaks and indicating significantly smaller crystallite sizes compared to the highly crystalline starting REO cake. The multi-REO NPs demonstrated remarkable antimicrobial activity, with agar-well assay inhibitory zones against Gram-positive (*Staphylococcus aureus* and *Bacillus cereus*) of 3.30 and 3.00 cm, respectively; against Gram-negative (*Escherichia coli*); and against fungal strains (*Candida albicans* and *Aspergillus niger*) of 2.5 and 2 cm, respectively. The nanoparticles were notably most effective against *S. aureus*.

## Abstract 2

Multi-rare earth oxide nanoparticles (∼29 nm) were green-synthesized using *Moringa oleifera* leaf extract. These NPs showed potent, broad-spectrum antimicrobial activity, offering a sustainable alternative against microbial pathogens.

## Graphical abstract

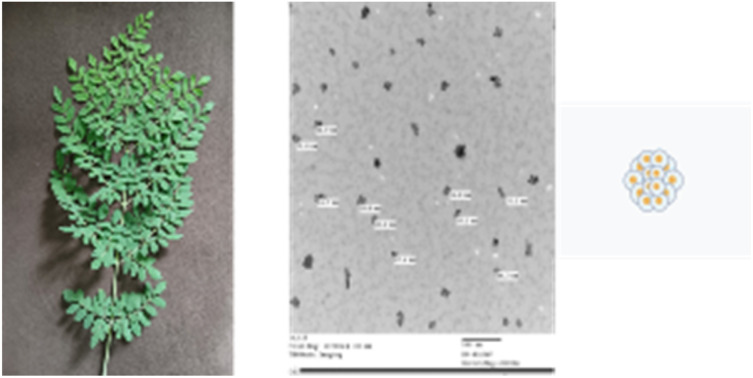

## Introduction

Rare-earth elements (REEs) are 17 chemically similar metallic elements that are vital to many cutting-edge applications in the rapidly developing fields of materials science and technology. The unusual electronic structure of these lanthanides, notably their 4f electron shells, permits precise manipulation of light, magnetism, and electrical fields at the atomic scale.^1^ This property places REEs as crucial components in numerous technical sectors, including renewable energy systems, electric vehicle manufacturing, enhanced medical imaging, and quantum computing.^2^

The transformation of REEs into nanoparticle form marks a significant achievement in harnessing their outstanding features. Hughes *et al.*^3^ revealed that “When converting these elements to the nanoscale, they acquire unique physical and chemical properties that distinguish them from their large-scale counterparts, mainly due to the increase in the ratio of surface-area-to-volume and the appearance of quantum confinement effects.” This nanoscale transformation opens new avenues for innovation in catalysis, optoelectronics, and targeted drug delivery systems, and it can be leveraged in biomedical research. For example, cerianite nanoparticles are being investigated as therapeutic agents for diseases associated with oxidative stress and inflammation, including cancer.^4^ The exact management of rare-earth oxide nanoparticles (REO NPs) has promise for advancing efficient solar energy devices, sophisticated data storage technologies, and revolutionary techniques in personalized medicine.^5^

The importance of rare-earth elements goes beyond their technological uses to encompass the geopolitical implications of their mining and refining.^6^ There is an increased global rivalry for resource security, intensified by the highly clustered geographical distribution of rare-earth element (REE) resources. This situation has increasingly integrated scientific research into broad economic and political strategies, as nations seek to secure access to these critical materials.^7^ As demand for these elements rises and environmental issues related to traditional extraction and processing methods become highly pressing, there is a prodigious push towards creating sustainable and eco-friendly synthesis methods for REE-based materials, especially those at the nanoscale.^8^ This reality, coupled with the diverse potential of these elements and their nanoparticles in materials science, engineering, environmental conservation, supply chain management, and sustainable development strategies, calls for a holistic approach to their study and applications,^9^ stimulating interdisciplinary innovation and fostering collaboration among previously distinct research areas.

**Fig. 1:**
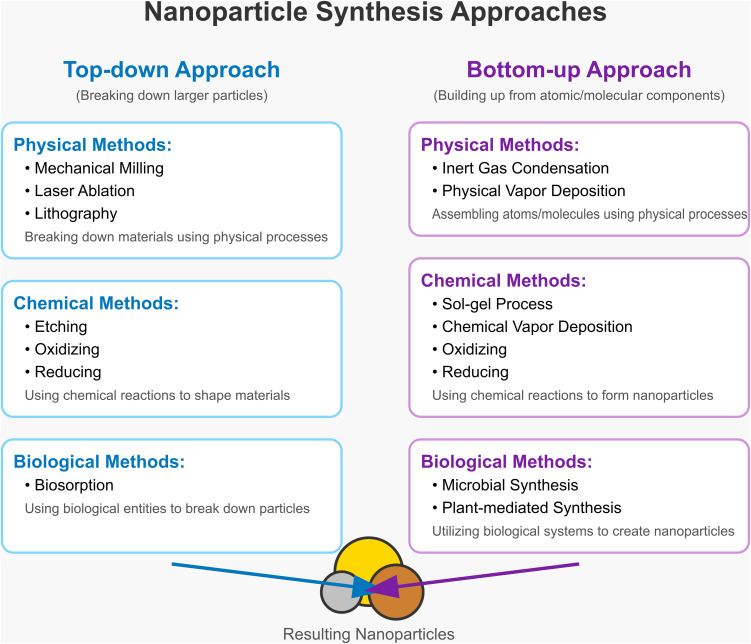
Representation of the two primary approaches for nanoparticle synthesis.

The synthesis of nanoparticles generally follows two distinct strategies: top-down and bottom-up approaches, which are illustrated in Fig. 1. These two general approaches to synthesis can be subdivided into physical, chemical, and biological methods. In the case of physical methods, it requires special equipment and setups. Conventional chemical synthesis methods, for example, usually involve severe reaction conditions, toxic reducing agents, and organic solvents, which can produce hazardous byproducts and raise important environmental concerns.^10^ In biological methods, plant-mediated nanoparticle synthesis has gained increasing attention as an environmentally safe nanoparticle production technique, now referred to as “green synthesis” or “biogenic synthesis” due to these concerns. These green synthesis methods generally utilize biological sources, including plant extracts and microorganisms such as bacteria and fungi, together with biomolecules that function as reducing and stabilizing agents, to produce nanomaterials through eco-friendly and non-toxic processes.^11,12^ Plant extract – based synthesis methods create rare-earth element nanostructures in a more sustainable and environmentally friendly way compared to traditional methods.^13^ The process of biosynthesis in plants relies on exploiting the natural capabilities of phytochemicals to act as both reductive and stabilizing agents, allowing the formation of rare-earth nanoparticles with manageable shapes and sizes.^14^ This technique not only minimizes harmful by-products associated with chemical operations but also boosts the bioavailability of these elements for numerous applications, including catalysis and medication administration.^15^ Hence, research into plant extracts for REEs production points towards a paradigm shift in green technologies in materials science and resource management.

The green approach presents multiple benefits, including the extensive availability and low-cost nature of plant materials, the natural biocompatibility of substances from plants and the potential for large-scale production with mild reaction conditions.^16^ Much research has shown the successful use of plant extracts in the biosynthesis of a variety of rare-earth element nanoparticles. This success is due to the richness of these extracts with bioactive secondary metabolic compounds, such as polyphenols and flavonoids, which act as reducing and stabilizing agents. For example, Karthikeyan *et al.*^17^ detailed the green synthesis of lanthanum oxide nanoparticles (La_2_O_3_ NPs) with a size range of 12 nm by the sol–gel approach. Arumugam *et al.*^18^ used *Gloriosa superba* L. leaf extract to produce cerium oxide nanoparticles (CeO_2_ NPs) with an average size of 24 nm. Subsequently, Monica Ahmad and Aishah Hasan^19^ used the sol–gel technique to biologically synthesize cerium oxide nanoparticles. Strieder *et al.*^20^ reviewed potential solutions to challenges facing eco-friendly methods, aiming to enhance the versatility and applicability of cerium oxide nanoparticles from botanical extracts. Miller *et al.*^21^ used a low-temperature one-pot polyol method to produce sesquioxide NPs and synthesize ultrasmall Eu_2_O_3_ NPs. Numerous NPs have been produced through the bio-reduction of biological compounds or their extracts.^22^ Nadeem *et al.*^23^ conducted a review on green synthesis methods for cerium oxide nanoparticles (CeO_2_ NPs) and explored their applications in antimicrobial treatments. Studies have shown that plant extracts have potential for REE nanoparticle synthesis, but research is limited regarding the biogenic synthesis of light rare-earth element (LREO) oxide nanoparticles with plant extracts.

The *Moringa* genus, from the Moringaceae family, features *Moringa oleifera* as its most cultivated species while belonging to the single-genus family within the Dicotyledoneae class. According to a previous study, there are 13 different species in this genus.^24^ This plant receives recognition for its rich phytochemical composition that features multiple bioactive compounds such as polyphenols, flavonoids, ascorbic acid and tannins.^25^*M. oleifera* extracts demonstrate strong reducing and antioxidant properties, which make them an excellent candidate for sustainable nanoparticle production.^26,27^ Research studies have demonstrated successful applications of *M. oleifera* extracts in the synthesis of various metal and metal oxide nanoparticles.^28^ Maheshwaran *et al.*^29^ developed irregular cubic and rectangular lanthanum oxide nanoparticles, Nuaman *et al.*^30^ conducted their research on nanoyttria (Mo–Y_2_O_3_ NPs), and Perumalsamy *et al.*^31^ reviewed the use of *M. oleifera* in the synthesis of metal nanoparticles. These studies highlight the potential of *Moringa oleifera* as a versatile biological resource for the environmentally friendly production of REE nanomaterials.

Recently, Titova *et al.*^32^ noted that the growing problem of global antibiotic resistance has necessitated accelerated research efforts to discover novel antimicrobial substances, leading to the emergence of REE nanoparticles as potential solutions. For instance, earlier studies have highlighted these concerns; a study by Zheng *et al.*^33^ revealed that nanoparticles made from rare-earth elements such as cerium oxide (CeO_2_), lanthanum oxide (La_2_O_3_), and europium oxide (Eu_2_O_3_) demonstrate strong antimicrobial effects against many bacterial and fungal species. Patil Maheshkumar *et al.*^34^ explained the functions of nanoceria through multiple potential mechanisms, which involve the generation of reactive oxygen species that cause oxidative stress and breakdown of microorganism membranes alongside essential cellular dysfunctions. REE nanoparticles produced through biological methods and plant extracts demonstrate varying antimicrobial properties compared to nanoparticles made by chemical synthesis, according to Shah *et al.*^35^ It is thought that biomolecules left on the surfaces of biologically produced nanoparticles may play a role in improving their compatibility with living tissues and modifying the way they interact with microbes, leading to a change in their antimicrobial properties.^36^ Maruthapandi *et al.*^37^ reported the effectiveness of plant source-synthesized carbon dots against *E. coli* and *S. aureus* at 1000 g mL^−1^.

Recent research has highlighted the *M. oleifera* plant as a versatile biological source for producing metal nanoparticles and their oxides.^26^ While its potential for synthesizing individual rare-earth element nanoparticles (REEs) is being explored, its application in creating nanoparticles from mixed rare-earth element sources (such as LREO) to leverage their potential for significant combined antimicrobial action has not been thoroughly investigated.

The importance of REEs in numerous technological and biomedical applications and their emerging role as nanoparticles emphasizes the rising significance of sustainable biogenic synthesis methods for REE and REO nanomaterials. Plant extracts from *M. oleifera* have shown potential as effective bioreductants and stabilizing agents in nanoparticle production. Although the antimicrobial characteristics of various REO NPs are well recognized, there is a pressing need for additional research dedicated to the biogenic synthesis of multi-REO NPs using *M. oleifera* and a comprehensive assessment of their antibacterial and antifungal capabilities. This study aims to resolve this information gap by exploring the environmentally friendly synthesis of LREO NPs and assessing their effectiveness as new antimicrobial agents for mitigating antibiotic resistance. To the best of our knowledge, this is one of the first studies to utilize a mixed rare-earth oxide precursor derived from a processed mineral concentrate (*i.e.*, processed Egyptian monazite) for the biogenic synthesis of multi-REO NPs using *Moringa oleifera* and to evaluate the synergistic antimicrobial effects of this nanoparticle mixture.

## Materials and methods

### Materials

Analytical grade nitric acid (HNO_3_) and ethanol were purchased from Sigma-Aldrich. Deionized (DI) water was used throughout the experiments. Light rare-earth oxide cake (LREO cake), previously prepared from Egyptian monazite mineral digestion by 98% sulfuric acid in hydrothermal autoclaves, were employed.^38^ The bulk elemental composition of this LREO cake was determined using inductively coupled plasma-optical emission spectrometry (ICP-OES; Prism ICP, Teledyne Leeman Labs, USA) following sample digestion in analytical grade HNO_3_, as per standard EPA guidelines.^39^

### Plant materials

Leaves of *Moringa oleifera* were collected from mature trees grown on a private farm in Faqus city, Sharkia Governorate, Egypt (approx. 30° 43′ 47′′ N, 31° 47′ 50′′ E). The plant identification is formally confirmed. The collected leaves were cleaned and then left to air-dry in the shade at room temperature until a constant weight was established. The dried leaves were ground into a fine powder using a laboratory mill to obtain a powder or material of a more uniform particle size, which is essential for consistent extraction efficiency. An analytical sieve machine, HAVER EML 200, was employed to sieve powder, which was then stored in airtight containers in the dark until further use.

### Preparation of *Moringa* extract

Dried *Moringa oleifera* leaf powder (5 g) was weighed and added to 50 mL of DI water. The mixture was stirred continuously at room temperature for 2 hours. The extract was then filtered through Whatman No. 1 filter paper to remove any solid residues. The obtained *Moringa* extract was stored at 4 °C for further use, following the method adapted from ref 40.

### Preliminarily phytochemical screening

Preliminary phytochemical screening of the prepared *M. oleifera* aqueous leaf extract was conducted following established qualitative methods^41,42^ to identify the presence of major classes of secondary metabolites (*e.g.*, flavonoids, tannins, saponins, and alkaloids). Detailed results of this screening are presented in the Results section.

### Green synthesis of LREO nanoparticles (LREO NPs)

**Fig. 2:**
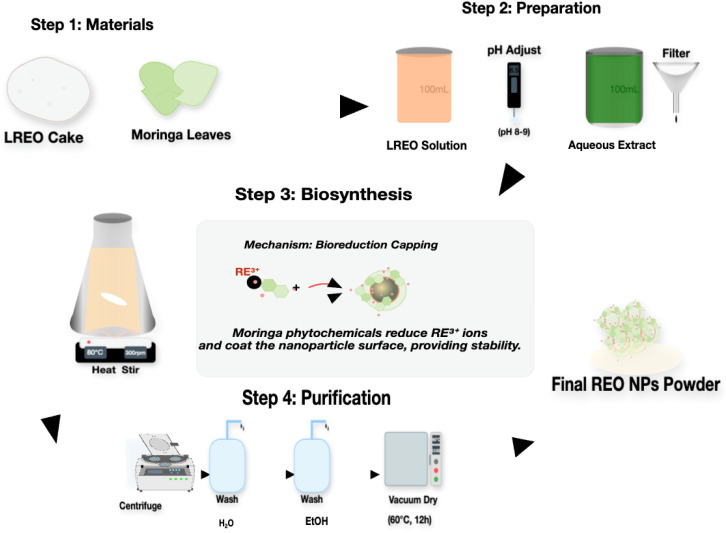
Schematic of the synthesis steps for REO NPs *via* the plant-mediated reduction method.

Light rare-earth oxides nanoparticles (LREO NPs) were synthesized using aqueous *M. oleifera* leaf extract as a bioreducing and capping agent (schematic shown in Fig. 2). Briefly, 1.0 g of LREO cake bulk powder was dispersed in 100 mL of DI water in an Erlenmeyer flask with the aid of continuous stirring for 15 min to ensure homogeneity. The pH of the aqueous LREO dispersion was adjusted to 8.0–9.0 by adding 1 M HNO_3_ dropwise under constant magnetic stirring. Then, 50 mL of *Moringa* extract was added to the solution. The reaction mixture was then heated to 80 °C and stirred continuously at 300 rpm for 2 hours. After cooling the mixture to room temperature, the LREO nanoparticle-containing precipitate was collected by centrifugation at 10 000 rpm for 20 minutes. To purify the precipitate, it was washed three consecutive times with deionized water and then once with absolute ethanol, with centrifugation repeated after each wash step. The final washed pellet was dried in a vacuum oven at 60 °C for 12 hours. The cooled LREO NPs powder was slightly ground and kept in a desiccator until characterization and further investigations.

### Characterization techniques

#### UV-visible spectroscopy (UV-vis)

The optical properties were studied by recording the absorption spectrum from 200 to 800 nm using a JASCO V-530 spectrophotometer. LREO NPs were dispersed in DI water at a concentration of 0.1 mg mL^−1^.

#### X-ray diffraction (XRD)

Crystalline structure analysis was performed using an Empyrean diffractometer (Malvern Panalytical) with Cu Kα radiation (*λ* = 1.5406 Å). Diffractograms were recorded over a 2*θ* range of 10° to 80° with a step size of 0.02° and a scan speed of 2° min^−1^. Phase identification was performed using X'Pert HighScore Plus software by comparison with standard JCPDS database files. The average crystallite size (*D*) of the starting LREO cake material was estimated from the line broadening of its most intense diffraction peak using the Scherrer equation: *D* = *Kλ*/(*β* cos *θ*), where *K* is the shape factor (0.94), *λ* is the X-ray wavelength (1.5406 Å), *β* is the full width at half maximum (FWHM) in radians, and *θ* is the Bragg angle.

#### Scanning electron microscopy and energy-dispersive X-ray spectroscopy (SEM-EDS)

Surface morphology and elemental composition were investigated using a Philips XL 30 Environmental Scanning Electron Microscope (ESEM) equipped with an EDS detector. For scanning electron microscopy (SEM) examination, the samples were prepared by fixing the nanoparticle powder onto an aluminum sample holder using double-faced carbon adhesive tape. The samples were then coated with a thin layer of carbon to increase their electrical conductivity and ensure high-quality imaging. SEM images were acquired at an accelerating voltage of 15 kV. EDS analysis was performed to confirm the presence of rare earth elements and to map their distribution.

#### Fourier transform infrared spectroscopy (FTIR)

Functional groups present on the nanoparticle surface were analyzed using a Thermo Scientific Nicolet Apex FTIR spectrometer equipped with OMNIC Paradigm Software. Spectra were recorded over the range of 4000–400 cm^−1^ with a resolution of 4 cm^−1^ by accumulating 32 scans. Samples were analyzed as KBr pellets.

#### Transmission electron microscopy (TEM)

Nanoparticle size, shape, and morphology were further examined using a JEOL JEM-1400Flash TEM operated at an accelerating voltage of 80 kV. Samples were prepared by dispersing a small amount of LREO NPs in a buffer (DI water/ethanol) using ultrasonication. A drop of the dispersion was deposited onto a carbon-coated copper grid and allowed to air-dry completely. Digital micrographs were captured at various magnifications. Image analysis was performed using ImageJ software. The diameters of at least 100 randomly selected, well-separated nanoparticles were measured. Particle size distribution histograms were generated using OriginPro software (Version 2024, OriginLab Corporation).

#### Dynamic light scattering (DLS)

The hydrodynamic diameter and particle size distribution of the synthesized multi-REO NPs in colloidal suspension were determined using dynamic light scattering (DLS). The sample was prepared by dispersing a small amount of nanoparticle powder in deionized (DI) water, followed by ultrasonication for 15 minutes to ensure a homogenous suspension. The analysis was performed using a particle size analyzer (Nicomp N3000, Particle Sizing Systems, Inc., Santa Barbara, CA, USA) at a fixed scattering angle of 90°. Measurements were conducted at 23 °C, and the instrument was equipped with a 632.8 nm laser. The data were analyzed using the NICOMP distribution algorithm to obtain the volume-weighted and number-weighted size distributions.

### Antimicrobial activity testing

The antimicrobial properties of biologically synthesized rare-earth oxide nanoparticles (REO NPs) and bulk rare-earth element oxides (REO cake) were evaluated using two distinct methods: the agar diffusion method and the broth dilution method. These assays tested the efficacy of the samples against five indicator microorganisms: *Candida albicans*, *Bacillus cereus*, *Aspergillus niger*, *Staphylococcus aureus*, and *Escherichia coli*.

#### Agar diffusion method

This methodology was modified from previous studies.^43,44^ Before the test, fresh cultures of the five microbial strains were prepared by growing them overnight. After that, the concentration of each culture was standardized to reach 10^6^ colony formation units per milliliter (10^6^ CFU per mL). Nutrient agar was used as the growth medium for bacterial strains (*B. cereus*, *S. aureus*, and *E. coli*), while potato dextrose agar was employed for the yeast (*C. albicans*) and fungal (*A. niger*) strains. The prepared cultures were inoculated into their respective molten agar media, which were then poured into sterile Petri dishes and allowed to solidify.

Suspensions of LREO NPs (100 μL) and bulk LREO cake (at concentrations of 20, 40, 200 and 2000 μg mL^−1^) were prepared in sterile distilled water. Wells (6 mm in diameter) were punched into the solidified agar. Aliquots (100 μL) of each test suspension were then added to these wells. For controls, ampicillin (10 μg mL^−1^) was used for bacteria, fluconazole (25 μg mL^−1^) was used for yeast and fungi (positive controls), and sterile distilled water served as the negative control. Plates were incubated for 24 hours at optimal temperatures (37 °C for bacteria and *C. albicans*; 25 °C for *A. niger*). The diameter of any resulting inhibition zones was measured in centimeters (cm). All tests were performed in triplicate, and results are reported as mean ± standard deviation.

#### Broth dilution method

The broth dilution method was employed to further quantify antimicrobial activity, following.^45^ For each of the five microorganisms, 100 μL of a standardized microbial suspension (10^6^ CFU per mL) was inoculated into 10 mL of either nutrient broth or potato dextrose broth for bacteria and fungi. Test samples (LREO NPs (multi-REO NPs) and bulk LREO cake) were introduced to achieve a final concentration of 3000 μg mL^−1^. Negative controls (broth with microorganism, no sample) and positive controls (broth with microorganism and a standard antimicrobial: ampicillin for bacteria; fluconazole for yeast/fungi) were included in each assay. After a 24 hour incubation at optimal growth temperatures, microbial growth was determined by measuring optical density (OD) at 600 nm with a spectrophotometer. The percentage inhibition of microbial growth was calculated using the following equation:Inhibition (%) = 100 − [(OD sample/OD negative control) × 100].

Experiments were conducted in triplicate for each microbial strain.

## Results and discussion

This investigation focused on the biosynthesis of multi-component rare-earth oxide nanoparticles (multi-REO NPs) using a light rare earth oxide cake (LREO cake) mixture as the precursor material. The LREO cake, comprising oxides of several lanthanides, served as the source for nanoparticle formation *via* a green synthesis approach mediated by *M. oleifera* leaf extract. The composition of the starting LREO cake material inherently influences the properties of the resulting nanoparticles. Elemental analysis of the LREO cake used in this work is presented in Table 1.

**Table 1:** Elemental composition of the light rare-earth oxide cake (LREO cake) precursor

Elements	Concentration (ppm)	Percentage (wt%)
La	8694	19.62
Ce	17 812	40.18
Pr	2182	4.92
Nd	12 222	27.56

The bioreduction and stabilization of the nanoparticles were facilitated using an extract derived from *M. oleifera* leaves. To identify potential participating biomolecules, phytochemical screening was performed on aqueous, methanolic, and petroleum extracts (Table 2). The aqueous extract, which was utilized for the nanoparticle synthesis described herein, tested positive for the presence of alkaloids, phenols, and flavonoids. In nanobiotechnology, *M. oleifera* extracts are frequently employed as sustainable precursors for synthesizing transition/post-transition metal nanoparticles due to their bioactive properties.^31^ These classes of compounds are known to possess reducing and chelating capabilities suitable for nanoparticle synthesis.^25,46^

**Table 2:** Phytochemical profile of *Moringa oleifera* leaf extracts^a^

Plant constituent	Aqueous extract	Methanolic extract	Petroleum ether extract
Alkaloids	+	−	−
Tannins	−	+	−
Phenols	+	+	−
Flavonoids	+	+	−
Steroids	−	−	+
Terpenoids	−	+	−

a (+) indicates presence; (−) indicates absence.

UV-vis spectroscopy (JASCO V-530 spectrophotometer, 200–800 nm) was used to characterize the formation of nanoparticles following the reaction between the LREO precursor and the aqueous *M. oleifera* extract. The resulting absorption spectrum (Table 3) displayed multiple features, including distinct peaks centered near 229 nm (assigned to π → π* transitions), 311 nm (attributed to *n* → π* or charge-transfer processes), 441 nm (indicative of d–d transitions/plasmon resonance), and 596 nm (suggestive of ligand–metal charge transfer). The absorption at 229 nm is consistent with charge-transfer transitions commonly observed in metal oxides. The peak at 311 nm may correspond to the optical band gap of the synthesized nanocrystalline oxides, potentially influenced by quantum confinement phenomena.^47^ The broad features at 441 nm and 596 nm are less readily assigned; the latter could potentially arise from characteristic f–f electronic transitions of constituent lanthanide ions, such as Nd, present in high concentration (Table 1),^48^ while the origin of the 441 nm peak warrants further investigation, possibly relating to surface defects or interactions with residual organic capping agents.

**Table 3:** UV-vis spectroscopy peak assignments of synthesized REO NPs

Peak position (nm)	Absorbance (au)	Proposed assignment	Band type
229	0.8	π → π* transition	Sharp
311	4.1	*n* → π* transition/charge transfer	Shoulder
441	4.3	d–d transition/plasmon resonance	Broad
596	2.8	Ligand–metal charge transfer	Resolved

The phytochemical profile of the aqueous extract (Table 2) supports its role in the synthesis. The presence of phenols and flavonoids, containing multiple hydroxyl groups, suggests that they act as primary reducing agents, converting the rare-earth element precursors into their oxide forms and subsequently capping the formed nanoparticles.^49,50^ Alkaloids may also participate in the reduction and stabilization process. These initial spectroscopic results suggest the successful formation of multi-REO NPs mediated by the *M. oleifera* aqueous extract. Further structural and morphological characterization is required to fully confirm the nanoparticle properties.

Fourier transform infrared (FTIR) spectroscopy was utilized to investigate the functional groups present in the *M. oleifera* leaf extract and the synthesized multi-component rare-earth oxide nanoparticles (multi-REO NPs). This analysis aimed to identify the biomolecules potentially involved in the reduction and capping process and to confirm the formation of the target metal oxides.

**Fig. 3:**
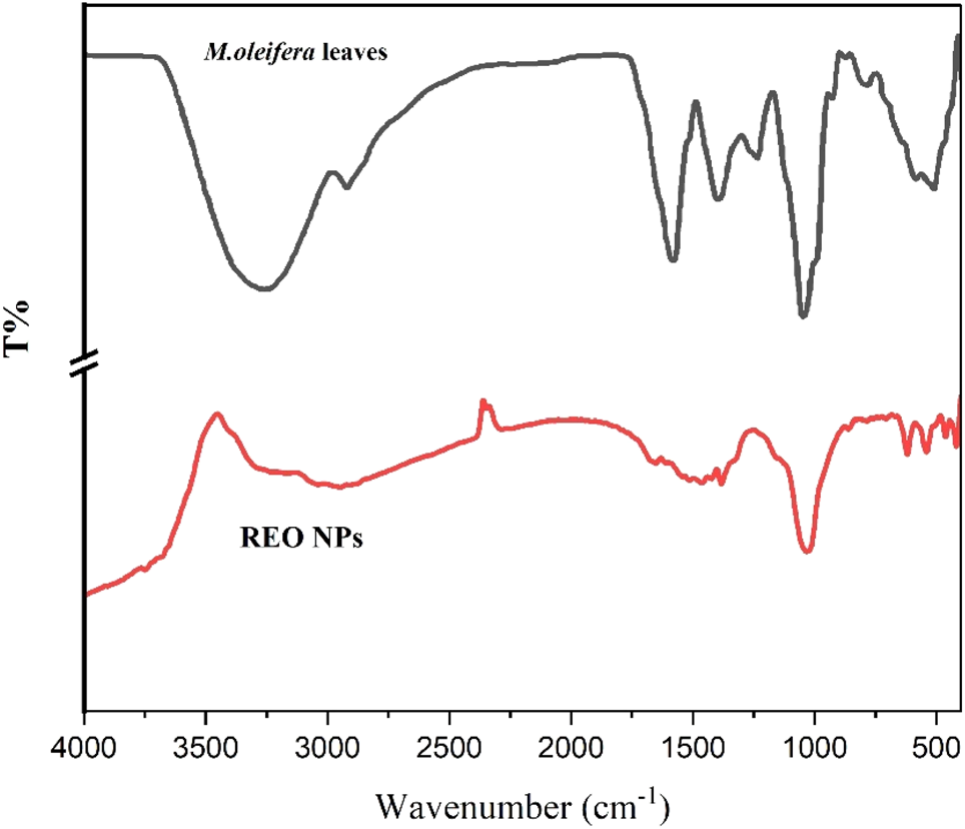
FTIR spectrum of *Moringa oleifera* leaves and REO NPs.

The FTIR spectrum of the *M. oleifera* leaf extract (as shown in Fig. 3) displayed characteristic absorption bands indicative of its rich phytochemical composition. A prominent broad band centered near 3362 cm^−1^ signals the presence of O–H stretching vibrations from hydroxyl and carboxylic groups prevalent in plant extracts, while peaks around 2921 cm^−1^ correspond to aliphatic C–H vibrations. Absorption at 1580 cm^−1^ likely arises from C=C stretching in aromatic rings, potentially from compounds like flavonoids or phenolic acids, and the band at 1395 cm^−1^ can be assigned to C–H bending modes. Significant absorption at 1045 cm^−1^ points to C–O stretching from alcohols, ethers, or polysaccharide components. These spectral features collectively confirm the presence of diverse biomolecules with functional groups such as hydroxyl, carboxyl, aromatic, and aliphatic moieties, making the extract a suitable source of reducing and stabilizing agents for nanoparticle synthesis.

Analysis of the spectrum of multi-REO NPs (also presented in Fig. 3) compared to the extract provides crucial insight into the biosynthesis process and nanoparticle–biomolecule interactions. The persistence of absorption bands corresponding to organic residues, specifically C–H stretches (*e.g.*, 2948.47, 1461.59, and 1382.83 cm^−1^) and C–O vibrations (1032.44 cm^−1^), confirms that organic components derived from the *Moringa* extract remain associated with the nanoparticle surface, effectively acting as capping or stabilizing agents. Furthermore, alterations or shifts observed in characteristic extract bands, particularly in the O–H (∼3362 cm^−1^), C=C (∼1580 cm^−1^), and C–O (∼1045 cm^−1^) regions, signify the direct involvement of these functional groups in the reduction of rare earth precursors and their binding to the nanoparticle surface. Crucially, the spectrum of NPs exhibits strong, distinct absorption bands in the low-wavenumber region at 619.50 cm^−1^, 540.57 cm^−1^, and 462.05 cm^−1^. These bands are characteristic of metal–oxygen (M–O) stretching vibrations within the rare earth oxide lattice. Specifically, the prominent absorption band observed around 462 cm^−1^ is attributed to the stretching vibration of the Ce–O bond in the cubic fluorite structure of CeO_2_ nanoparticles, in agreement with ref. 51. The other bands in the 500–620 cm^−1^ region likely correspond to M–O bonds involving other rare earth elements, such as La–O and Nd–O, confirming the formation of a mixed-oxide nanocomposite, consistent with ref. 52. Overall, the FTIR data decisively support the plant-mediated biosynthesis of multi-REO NPs, demonstrating both the formation of the metal oxide core and concurrent surface functionalization by bioactive molecules derived from the *M. oleifera* extract.

**Fig. 4:**
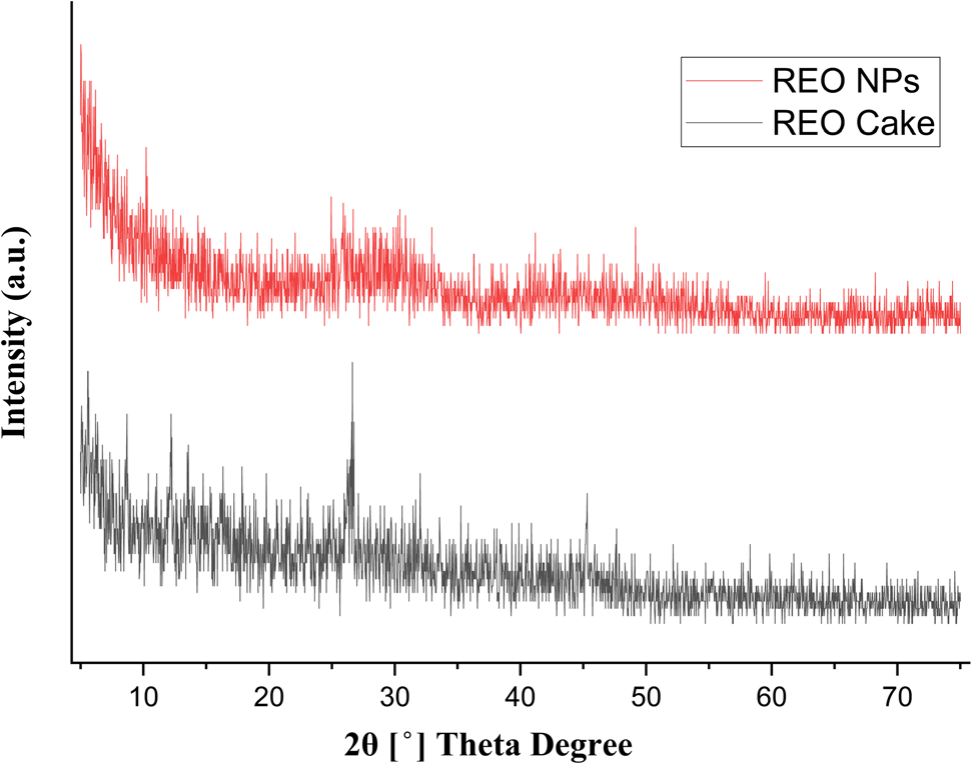
X-ray diffraction pattern of the synthesized REO NPs and REO cake.

To determine the crystalline structure, phase composition, and average crystallite size of the precursor material and the synthesized nanoparticles, X-ray diffraction (XRD) analysis was performed. The XRD analysis demonstrates notable structural variations between the rare earth oxide cake and biologically synthesized nanoparticles. The XRD pattern of the REO cake (Fig. 4) demonstrates complexity through multiple distinct sharp peaks on a broad background, which shows that the sample is a heterogeneous mixture of different rare earth oxide phases with varying levels of crystallinity. Industrial rare earth concentrates with multiple elements in bulk form exhibit well-crystallized phases and large crystallite sizes, which produce intense peaks in the low-angle region between 10 and 15° 2*θ*.

Biologically synthesized REO NPs (Fig. 4) present diffraction peaks that are broad and less intense, demonstrating the characteristics features of nanoscale material, where peak broadening correlates with small crystallite sizes as determined by the Scherrer equation. Peak broadening indicates that the nanoparticle synthesis yielded crystallites much smaller than those found in the original material. The nanoparticle pattern demonstrates a reduced number of distinct reflections along with a simplified peak structure, which hints that the biological synthesis may have driven selective phase formation or purification. The nanoparticle sample retains broadened crystalline peaks, which shows that the biological synthesis preserved the essential crystal structure of the rare earth oxides while reducing particles to nanoscale dimensions. The broad diffraction peaks observed at 2*θ* values of approximately 28.5°, 47.5°, and 56.3° can be indexed to the (111), (220), and (311) crystallographic planes of the cubic fluorite structure of CeO_2_, which is the primary component of the multi-REO NPs. This identification is consistent with standard data from JCPDS card no. 34-0394. The broadening of the peaks is a characteristic feature of nanomaterials, confirming the small crystallite size. Comparative analysis reveals that the biological synthesis approach transformed bulk REO cake into nanocrystalline materials while retaining essential structural features, which proves that the bio-mediated synthesis method is effective for creating rare earth oxide nanoparticles.

**Fig. 5:**
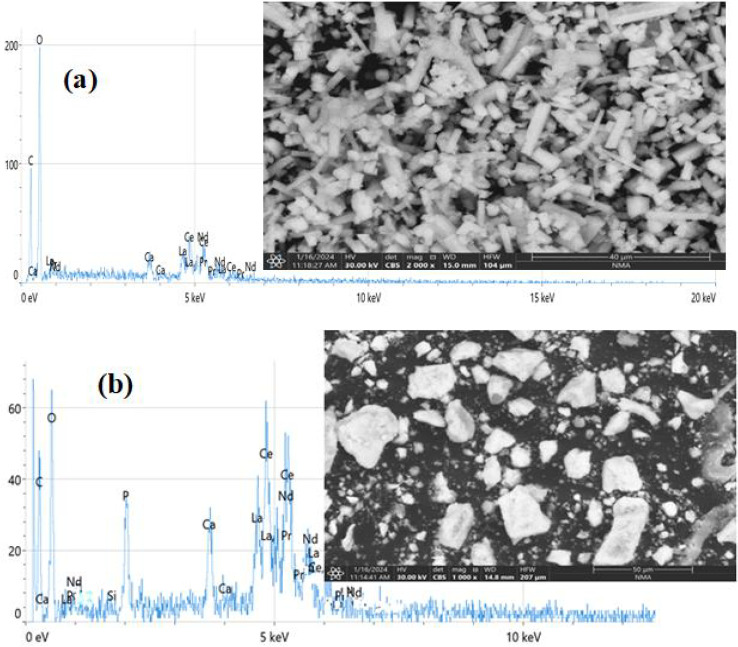
SEM image and EDS spectra of (a) REO cake and (b) REO NPs.

The surface morphology, particle shape, and elemental composition of the samples were investigated using scanning electron microscopy (SEM) coupled with energy dispersive X-ray spectroscopy (EDS).The morphology and elemental composition of the synthesized nanoparticles were examined using scanning electron microscopy (SEM) and energy-dispersive X-ray spectroscopy (EDS). SEM imaging (Fig. 5) was used to observe the bulk material and the synthesized NPs. In Fig. 5b, the formation of nanoparticle aggregates composed of roughly quasi-spherical primary particles is evident. The estimated diameter of these primary particles ranged from approximately 80 to 300 nm. In accordance with typical observations in nanomaterials, the average particle size measured by scanning electron microscopy (SEM) was larger than the average crystallite size determined from X-ray diffraction (XRD) data. This discrepancy arises because SEM measures particle aggregates, whereas XRD determines the size of individual crystallites. SEM shows the bulk, aggregated morphology of the dried powder, whereas TEM analysis confirms the true nanoscale nature of the primary particles.

Elemental analysis was performed using EDS (Fig. 5b). The spectrum confirmed the presence of the anticipated rare earth elements—cerium (Ce), lanthanum (La), neodymium (Nd) and praseodymium (Pr)—along with a strong oxygen (O) signal, consistent with the formation of rare earth oxides. A significant carbon (C) signal was also detected, attributable to residual organic material from the *M. oleifera* extract, acting as a capping agent.

Quantitative EDS analysis provided the elemental composition: O (53.2%), C (20.2%), phosphorus (P, 7.3%), Ce (7.0%; 26.4 wt%), La (3.3%; 12.2 wt%), Nd (2.7%; 10.4 wt%) and Pr (1.3%, 5.0 wt%). Trace amounts of silicon (Si) and calcium (Ca) were also detected. The high proportions of oxygen and carbon support the presence of both the metal oxide core and a substantial organic surface layer derived from the plant extract.^53^ The relative abundance of the detected REEs (Ce > La > Nd > Pr) reflects their initial concentrations in the precursor material, although direct stoichiometric determination of the oxide formula is complicated by the dominant C and O signals from the organic component.

The presence of a significant amount of phosphorus (7.3 at%) was unexpected based solely on the LREO cake precursor and *Moringa* extract; its origin requires further investigation but may suggest the involvement of phosphate species, potentially from the biological source or trace contaminants, in complexation or stabilization.^54^ The detection of multiple rare earth elements (Ce, La, Nd and Pr) within the analyzed areas confirms the formation of mixed-oxide nanoparticles, reflecting the composition of the LREO cake precursor. The heterogeneous elemental distribution corroborates the formation of mixed-REE oxide nanoparticles, reflecting the composition of the original REO precursor. This elemental fingerprint not only confirms the successful synthesis of REO NPs but also provides insights into the complex interplay between plant extract components and REO ions during the reduction process. Such intricate interactions underscore the need for comprehensive characterization in nanoecotoxicology studies, as emphasized by ref. 55. The EDS analysis provides compelling evidence for the successful synthesis of multi-rare-earth oxide nanoparticles through a green synthesis approach using *Moringa* leaf extract. The detection of lighter lanthanides (La–Gd) suggests a preferential reduction of these elements during biogenic synthesis, a phenomenon previously noted in green synthesis approaches.^56^

In conclusion, the combined SEM and EDS analyses confirm the synthesis of aggregated, quasi-spherical multi-component rare earth oxide nanoparticles with primary particle sizes in the 30–80 nm range. The elemental composition verifies the incorporation of La, Ce, Nd, and Pr, alongside significant carbon and phosphorus content associated with the organic materials used in the synthesis.

**Fig. 6:**
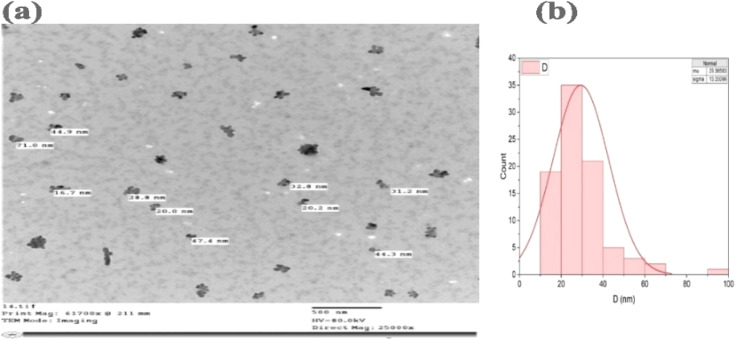
(a) TEM micrograph of synthesized REO NPs and (b) their particle size distribution histogram.

To obtain high-resolution images of the individual nanoparticles and accurately determine their size, shape, and size distribution, transmission electron microscopy (TEM) was utilized. TEM analysis was conducted to investigate the morphology and size distribution of the synthesized multi-REO NPs. The TEM image in Fig. 6a shows a distribution of nanoparticles with diverse morphologies, including spherical, irregular, and some elongated shapes, with an average particle size of 29.36 nm, as illustrated in the histogram (Fig. 6b). The histogram generated using Origin software shows a unimodal distribution, indicating a relatively homogeneous sample. The standard deviation of the particle sizes is 13.35 nm, suggesting a moderate degree of size variability within the sample.

**Fig. 7:**
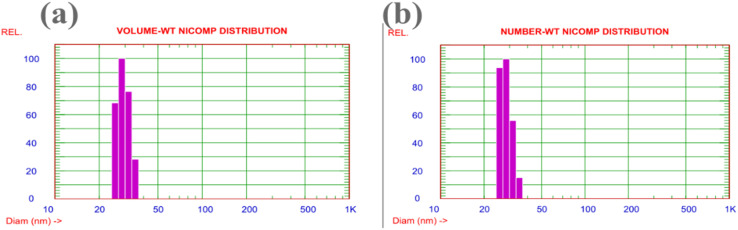
Dynamic light scattering (DLS) analysis of the synthesized multi-REO NPs. (a) Volume-weighted and (b) number-weighted particle size distribution, showing a mean hydrodynamic diameter of 29.3 nm.

To assess colloidal stability and determine the effective size of the nanoparticles in an aqueous environment, which is crucial for their potential biological applications, dynamic light scattering (DLS) analysis was conducted. The DLS results (Fig. 7) provide compelling confirmation of the size and stability of the biogenically synthesized nanoparticle. Notably, the mean hydrodynamic diameter of 29.3 nm (Fig. 7a) exhibits an exceptional correlation with the average physical diameter of 29.36 nm measured by TEM. While a slightly larger hydrodynamic diameter is often expected due to solvent and capping agent layers, this remarkable agreement suggests the formation of highly compact nanoparticles with a minimal, tightly bound organic surface layer derived from the *Moringa* extract. Furthermore, the unimodal and narrow size distribution revealed by the NICOMP analysis (Fig. 7b) confirms that the sample is highly monodisperse, which is a key indicator of an efficient and controllable synthesis process. Crucially, these findings demonstrate excellent colloidal stability, ensuring that the nanoparticles remain well-dispersed in an aqueous medium. This validates the results of the antimicrobial assays, confirming that the observed biological activity originates from individual nanoparticles rather than from undefined agglomerates.

### Antimicrobial study

The aim of this study was to investigate the antimicrobial activity of biologically synthesized rare earth oxide nanoparticles (multi-REO NPs) and bulk rare earth oxides (REO cake) against five microorganisms: *Candida albicans*, *Bacillus cereus*, *Aspergillus niger*, *Staphylococcus aureus*, and *Escherichia coli*. The study employed agar diffusion to measure inhibition centers in cm and broth dilution to assess the percentage of growth inhibition, thus providing a robust evaluation of their antibiotic potencies.

#### Agar diffusion method

**Fig. 8:**
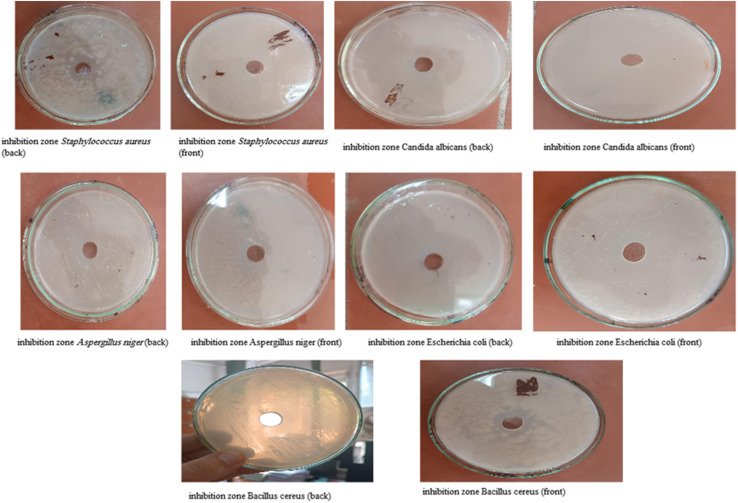
Effects of different concentrations on the inhibition zone diameter (MIC) for multi-NPs.

The agar diffusion assay demonstrated that muli-REO NPs possess robust antimicrobial activity across all tested microorganisms. As detailed in Table 4 and Fig. 8, inhibition zones for multi-REO NPs ranged from 2.00 cm against *Aspergillus niger* to 3.30 cm against *Staphylococcus aureus*, reflecting broad-spectrum effectiveness. Notably, *S. aureus* and *Bacillus cereus* (3.00 cm) exhibited the largest inhibition zones, underscoring the potency of nanoparticles against Gram-positive bacteria.

**Table 4:** Inhibition zones of REO nanoparticles (multi-REO NPs) against various microorganisms, measured using the agar diffusion method^a^

Multi-REO NPs (μg mL^−1^)	Inhibition zone of microbial growth (cm)				
	Microorganisms				
	*S. aureus*	*E. coli*	*C. albicans*	*B. cereus*	*A. niger*
100	3.30	2.80	2.50	3.00	2.00

a Inhibition zones were measured after 24 hours of incubation at optimal growth temperatures for each microorganism. Multi-REO NPs were tested at a concentration of 100 μg mL^−1^.

**Fig. 9:**
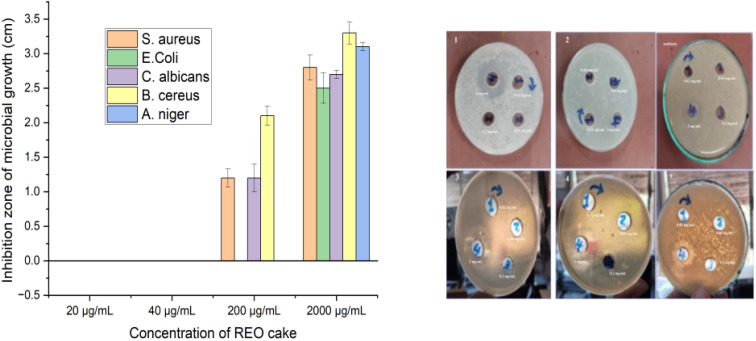
Antimicrobial activity of bulk REO cake at different concentrations from 1 to 4 (20, 40, 200, and 2000 μg mL^−1^) measured using the agar diffusion method. Data are presented as mean inhibition zone (cm) ± standard deviation (*n* = 3).

Conversely, bulk REO cake displayed concentration-dependent activity (Table 4; Fig. 9). At low concentrations (0.02 and 0.04 mg mL^−1^), no inhibition was observed. At 0.2 mg mL^−1^, inhibition zones emerged for *S. aureus* (1.20 ± 0.13 cm), *C. albicans* (1.20 ± 0.20 cm), and *B. cereus* (2.10 ± 0.14 cm), while *E. coli* and *A. niger* remained unaffected. At the highest concentration (2 mg mL^−1^), inhibition zones increased significantly, ranging from 2.50 ± 0.22 cm (*E. coli*) to 3.30 ± 0.16 cm (*B. cereus*), with *A. niger* showing a notable 3.10 ± 0.06 cm. These findings indicate that bulk REO cake can outperform REO NPs at elevated concentrations, particularly against *A. niger*.

#### Broth dilution method

**Fig. 10:**
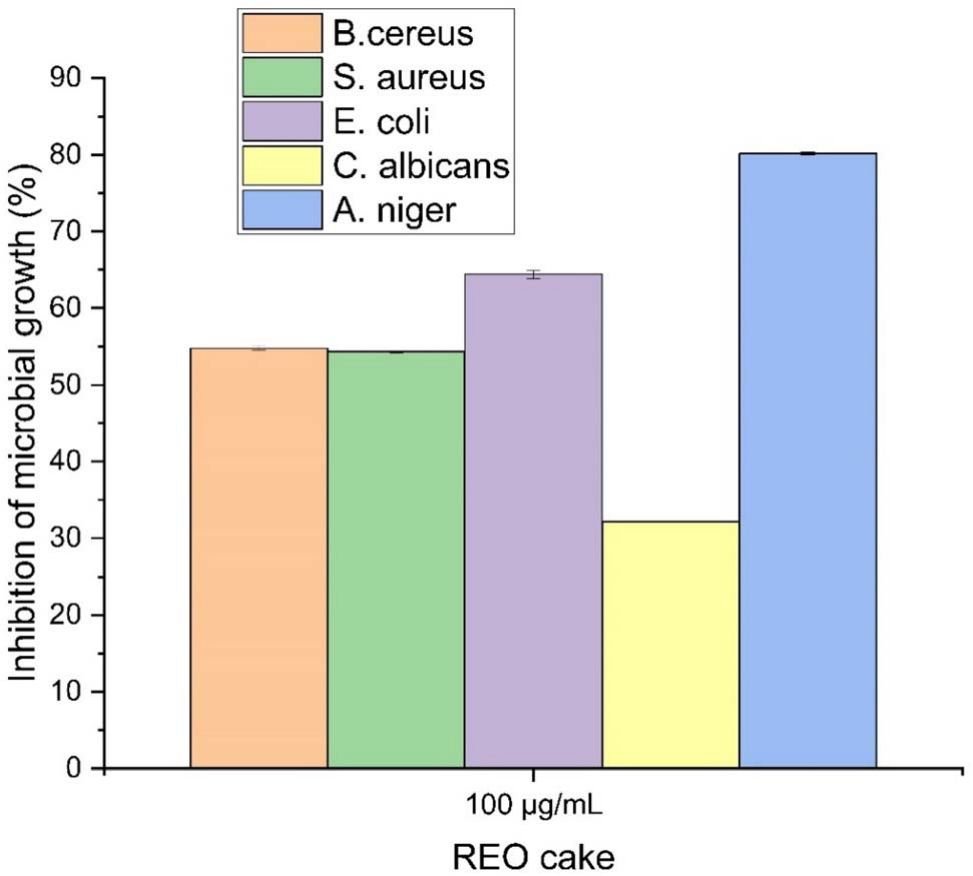
Percentage of microbial growth inhibition by bulk REO cake (3000 μg mL^−1^) measured using the broth dilution method. Data are presented as mean ± standard deviation (*n* = 3).

In the broth dilution assay, LREO NPs (multi-REO NPs) were tested solely against *Candida albicans*, achieving a growth inhibition of 60.71% (Table 5). For bulk REE oxides, inhibition percentages varied across all five microorganisms (Fig. 10). The highest inhibition was observed against *A. niger* (80.18% ± 0.19%), followed by *E. coli* (64.41% ± 0.55%), with the lowest against *C. albicans* (32.13% ± 0.07%). The superior inhibition of *C. albicans* by LREO NPs compared to bulk LREO cake suggests enhanced efficacy of the nanoparticle formulation in liquid media. However, the absence of broth dilution data for LREO NPs against the other microorganisms limits a complete comparison.

**Table 5:** Growth inhibition of *Candida albicans* by multi-REO NPs, measured using the broth dilution method^a^

Sample	Concentration (μg mL^−1^)	Inhibition of microbial growth (%)
REO NPs	3000	60.71

a Inhibition percentage was calculated using the following formula: inhibition (%) = 100 − [(OD sample/OD negative control) × 100], where, OD is the optical density measured at 600 nm after 24 hours of incubation.

The most significant result of this study is that, even though both the nanoparticle (REO NPs) and the bulk form of the rare earth oxides do possess antimicrobial activity, the nano-formulation shows superior performance, especially at lower concentrations. Findings obtained from the agar diffusion test undoubtedly confirm that REO NPs exhibit broad-spectrum and extremely effective antimicrobial activity at a low concentration of 100 μg mL^−1^. By contrast, the amounts of bulk REO cake were much higher (usually 2000 μg mL^−1^) to generate inhibition zones of similar size in size.

The experiment also showed that the effectiveness of the nanoparticles varied depending on the test conditions. In the case of *C. albicans*, despite the 30 times greater concentration (3000 μg mL^−1^) used in the broth dilution assay (compared to 100 μg mL^−1^ on agar), REO NPs showed only moderate inhibition (maximum 60.71% inhibition), though they were highly potent at 100 μg mL^−1^ on agar. This suggests that derivatives in the liquid broth greatly interfere with the antimicrobial activity of the nanoparticles. The possible reasons for this could be attributed to the physical and chemical reactions inside the broth. Despite this reduced efficacy in broth, the superiority of the nano-formulation is still evident. When directly compared against *C. albicans* at the same high concentration of 3000 μg mL^−1^, REO NPs (60.71% inhibition) were nearly twice as effective as bulk REO cake (32.13% inhibition). This confirms the inherent advantage of the nanoparticle structure even in a challenging environment.

The antimicrobial efficacy of multi-REO NPs stems from synergistic pathways.^57^ Reactive oxygen species (ROS), such as superoxide and hydroxyl radicals, dominate antimicrobial activity,^58,59^ and some recent evidence suggests that rare earth ions may inactivate efflux pumps.^60^ REO NPs often carry a positive surface charge, facilitating electrostatic attraction to negatively charged bacterial cell walls. This direct contact can disrupt membrane integrity, increase permeability, and cause cytoplasmic leakage, further contributing to microbial killing.^61^

The present study shows the promising antimicrobial potential of these multi-REO NPs, consistent with previous findings.^62^ The fact that they are active at low doses in a solid-state diffusion assay is a particularly remarkable finding. However, for applications in liquid environments, agglomeration and interactions with medium components must be taken into account. This means that the application potential could be unlocked by future studies, for example, surface modification or stabilization agents.

## Conclusion

This study presents a novel and eco-friendly biogenic approach for synthesizing multi-component rare-earth oxide nanoparticles (multi-REO NPs) from Egyptian monazite concentrate cake using *Moringa oleifera* leaf aqueous extract. The research validates this green synthesis approach by producing stable, well-characterized REO NPs with potent antimicrobial properties against Gram-positive bacteria (*Staphylococcus aureus* and *Bacillus cereus*, inhibition zones of 3.30 and 3.00 cm, respectively), Gram-negative bacteria (*Escherichia coli*), and fungal strains (*Candida albicans* and *Aspergillus niger*, inhibition zones of 2.5 and 2 cm, respectively), with notable effectiveness against *S. aureus*. Using *M. oleifera* extract as both a reducing and capping agent, this method tackles environmental issues related to traditional chemical synthesis and provides a non-toxic, renewable alternative. Both microscopic and spectroscopic investigations validated the synthesis of accurate rare-earth oxide nanoparticles (REO NPs) with an average particle size of 29.36 nm, which demonstrate the dual operational features of the extract.

The synthesized nanoparticles possess exceptional antimicrobial activity against multiple pathogens, while the REO cake shows good antimicrobial potential against various microorganisms, although at elevated concentrations. These findings indicate beneficial uses in healthcare services together with environmental applications, which can help combat antibiotic resistance problems. Also, research establishes a useful relationship between mineral resource use and nanotechnology through its discovery of sophisticated nanomaterials sourced from Egyptian processed monazite concentrates. The approach developed in this research has the potential to be expanded for synthesizing nanoparticles from additional rare-earth elements to promote widespread acceptance of environmentally friendly biosynthetic methods.

## Conflicts of interest

There are no conflicts to declare.

## Data Availability

The raw data supporting the conclusions of this article are available from the corresponding author upon reasonable request. This includes: UV-Vis spectroscopy data, FTIR spectral data, XRD diffraction patterns and analysis, SEM-EDS elemental mapping and quantitative data, TEM images and particle size distribution measurements, Antimicrobial assay raw data (zone measurements and optical density readings), Phytochemical screening results, Statistical analysis files. Some data may be subject to confidentiality agreements and cannot be made publicly available.
